# Prognostic Validity of the Eighth Edition of the U.S. Joint Committee on Cancer TNM Staging System for Pancreatic Adenocarcinomas: An Analysis of 214 Patients at a Spanish Center

**DOI:** 10.3390/cancers17111890

**Published:** 2025-06-05

**Authors:** A. M. Colino-Gallardo, M. J. Fernández-Aceñero, M. de la Torre-Serrano, J. Vega-González, M. P. Díaz-Suárez, J. Martínez-Useros

**Affiliations:** 1 Department of Surgical Pathology, Hospital Infanta Sofía, San Sebastián de los Reyes, 28702 Madrid, Spain; amcolinog@gmail.com; 2 Grupo de Investigación en Tumores Digestivos, Hospital Clínico San Carlos, IdiSSC, 28040 Madrid, Spain; montsedelatorre14@gmail.com; 3 Department of Surgical Pathology, Hospital Clínico San Carlos, 28040 Madrid, Spain; jesusvegagon@gmail.com (J.V.-G.); mariaadiaazsuaarez@gmail.com (M.P.D.-S.); 4 Department of Legal Medicine, Psychiatry and Surgical Pathology, Complutense University of Madrid, 28040 Madrid, Spain; 5 Translational Oncology Division, OncoHealth Institute, FIIS-Fundacion Jimenez Diaz, 28040 Madrid, Spain; javier.museros@quironsalud.es; 6 Physiology Area, Department of Basic Health Sciences, Health Sciences Faculty, University Rey Juan Carlos, Alcorcon, 28933 Madrid, Spain

**Keywords:** cancer, pancreas, TNM classification

## Abstract

**Introduction**: Accurate staging is essential in pancreatic adenocarcinoma due to its aggressive nature and poor prognosis. The 8th edition of the AJCC TNM staging system introduced changes in tumor size criteria and nodal classification. This study compares the prognostic performance of the 7th and 8th editions in resected patients. **Material and Methods**: A retrospective analysis was conducted on 214 patients with pancreatic adenocarcinoma who underwent curative surgery. TNM staging was assigned according to both AJCC editions. Kaplan–Meier analysis and multivariate Cox regression, stratified by adjuvant therapy, were used to assess disease-free survival (DFS) and overall survival (OS). **Results**: The 8th edition TNM staging was significantly associated with lower risk of recurrence, with TNM stages I and II independently predicting better DFS (*p* < 0.05). In contrast, the 7th edition TNM stage I remained the only independent predictor of OS (HR = 0.376; *p* = 0.023). Reclassification between editions altered stage distribution, particularly within stage II. **Conclusions**: The 8th edition improves early recurrence stratification, while the 7th edition retains stronger prognostic value for overall survival. Both systems offer complementary insights, supporting outcome-specific staging use.

## Simple Summary

Pancreatic cancer is one of the deadliest cancers, and accurate staging systems are essential to predict how the disease will progress and to guide treatment decisions. Over time, the staging system for pancreatic cancer has evolved, particularly between the seventh and eighth editions of the classification used by most specialists. This study evaluates how these two staging systems differ in real-world patients and whether one offers better predictions of disease progression and survival. It also investigates whether the number of affected lymph nodes or their proportion in relation to the total examined could improve prognostic accuracy. Our findings suggest that the new edition offers better categorization for predicting tumor progression but does not significantly improve survival prediction. These insights may help clinicians and researchers to better understand how to refine staging systems and identify high-risk patients more effectively.

## 1. Introduction

Pancreatic cancer remains a major global health concern, with its incidence steadily increasing in recent years. It is currently the fourth-leading cause of cancer-related deaths, with a 5-year survival rate of only 8% in the United States, and it is expected to be the second-leading cause of cancer death in 2030 [1,2,3,4,5]. Several factors are related to the prognostic outcome of patients with pancreatic cancer, including TNM stage, histologic differentiation, tumor size, lymph node (LN) status, and age [4]. Surgical treatment is the only curative method, but fewer than 20% of cases can be operated on when diagnosed [2,5]. Furthermore, the recurrence rate reaches 60–80% even with surgery, so a robust staging system with strong prognostic value is crucial for guiding treatment decisions and follow-up strategies [1,2,6,7]. Therefore, a clinically applicable and straightforward staging classification that effectively stratifies patients into prognostic groups is essential [1,6,8].

The seventh edition of the TNM staging system (T: primary tumor, N: regional lymph nodes, M: distant metastasis) for pancreatic cancer, developed by the American Joint Committee on Cancer (AJCC), was published in 2010 and has since been widely used in clinical practice (Table 1 and Table 2) [9]. However, concerns have been raised regarding the reproducibility of this staging system, particularly in defining the T stage, due to the ambiguity of the term “extension beyond the pancreas.” Extrapancreatic extension is difficult to determine and it is challenging to determine histopathologically because the pancreas does not have a capsule. The distinction between the pancreas and extrapancreatic tissue is often obscured by fibrosis, which may lead to inconsistent interpretations among pathologists [1,6,8,10,11,12,13].

Similarly, the N classification in pancreatic cancer staging has also faced criticism. The seventh edition only included one node-negative category (N0) and one node-positive category (N1), without differentiating between cases with limited versus extensive lymph node involvement. The lack of an N2 category for patients with multiple positive nodes has been regarded as a limitation, and, at the same time, recent studies have shown that not only the presence of nodal involvement but also the number of positive lymph nodes predicts survival outcomes [1,6,8,10,11,12,13].

In response to these concerns, the eighth edition of the AJCC staging system for pancreatic cancer published in 2016 incorporated two major modifications:

- The T stage now considers tumor size instead of tumor extension beyond the pancreas;
- The N stage has been revised to introduce a new N2 category, where N1 represents 1–3 positive lymph nodes, and N2 includes cases with ≥4 positive lymph nodes [1,11,12].

This study aims to compare the seventh and eighth editions of the AJCC TNM staging system in pancreatic adenocarcinoma, using a dataset of 214 patients operated on with a curative intent in a single Spanish tertiary hospital.

## 2. Materials and Methods

### 2.1. Data Collection

This study retrospectively analyzed all the patients diagnosed with pancreatic adenocarcinoma who underwent surgical resection between 2000 and 2022 at the San Carlos University Clinical Hospital in Madrid. This hospital is a reference center for pancreatic cancer management in Madrid and has a team of surgeons with extensive expertise in pancreatic cancer surgery. Medical records were reviewed to obtain relevant clinical and pathological data. Tumors of the biliary tract located within the pancreas were also included, as they share similar clinical behavior and management, and histological confirmation was required for all patients, ensuring that only cases with a definitive diagnosis were included. The exclusion criteria for the study were tumors located exclusively outside the pancreas (such as ampullary tumors), diagnosis based only on cytology without resection, and non-adenocarcinoma histology (e.g., neuroendocrine tumors).

To ensure a comprehensive dataset, clinicopathological variables were retrieved from the databases of the Pathology Department (Patwin and AnaPath). These included demographic information, tumor characteristics, surgical details, and follow-up data. The demographic variables collected encompassed age, sex, and the date of surgery. Tumor characteristics were meticulously recorded, including tumor size (defined as the largest macroscopic diameter), tumor location, histological grade, and the presence of lymphovascular and perineural invasion. Given the relevance of lymph node involvement in pancreatic cancer prognosis, information regarding lymph node status was also collected, including the total number of nodes examined and the number of positive nodes. All the specimens were managed following the same standardized protocol by a group of gastrointestinal pathologists.

Particular attention was given to the TNM classification of each case. Staging information was recorded according to both the 7th and 8th editions of the American Joint Committee on Cancer (AJCC) classification system (Table 3), allowing for a direct comparison between staging criteria and their impact on clinical outcomes. The study also considered whether patients received adjuvant therapy, defined as any postoperative systemic treatment, including chemotherapy and/or radiotherapy.

Follow-up data were included to assess disease recurrence and survival outcomes. As all patients were operated on with a curative intent, the presence of tumor relapse/progression was recorded, specifying the type of progression (local recurrence, distant metastasis, or both), along with the date on which progression was first detected, to measure the disease-free survival (DFS) in months. This study also documented whether the patient was alive or deceased at the time of data collection, recording the date of death or the date of last known contact. The overall survival time (OS) was calculated for each patient, from the date of surgery to the last recorded follow-up or death in months.

The study protocol was approved by the Institutional Review Board (IRB) of the San Carlos University Clinical Hospital in Madrid (protocol number 23/584-E_Tesis), ensuring compliance with ethical standards and patient confidentiality guidelines.

### 2.2. Statistical Analysis

All collected data were anonymized and securely stored in a Microsoft Excel database, which was subsequently imported into IBM SPSS Statistics for Mac, version 27.0 (IBM Corp., Armonk, NY, USA) for statistical analysis.

Descriptive analyses were performed to summarize both quantitative and categorical variables. Continuous variables were assessed for normality using the Shapiro–Wilk test, and data were expressed as mean. If they follow a normal distribution, they are indicated by the mean and the range, which includes the minimum and maximum values if they followed a normal distribution (*p* > 0.05). Categorical variables were described using absolute frequencies and percentages.

To examine changes in staging categorization between the 7th and 8th editions of the TNM classification, a comparative analysis was conducted. The T, N, and TNM classifications from both editions were systematically evaluated to determine how modifications in staging criteria influenced disease classification and prognosis. Special attention was given to differences in the definition of T stage, where tumor size replaced the criterion “extension beyond the pancreas”, as well as the introduction of the N2 category for cases with ≥4 positive lymph nodes.

The relationship between TNM staging and disease progression was also assessed. The association between TNM staging (7th and 8th editions) and clinical outcomes was analyzed using Chi-squared tests (χ^2^) to determine whether staging modifications affected progression patterns and overall survival. The following comparisons were made: T stage (7th vs. 8th edition); N stage (7th vs. 8th edition); TNM stage (7th vs. 8th edition); TNM stage (7th edition) vs. disease progression; TNM stage (7th edition) vs. overall survival; TNM stage (8th edition) vs. disease progression; and TNM stage (8th edition) vs. overall survival. A *p*-value < 0.05 was considered statistically significant in all comparisons. These analyses were used to assess the general distribution of clinical outcomes across TNM categories, rather than to provide adjusted prognostic modeling. Variables with missing data—such as perineural invasion or lymph node status—were described in the overall cohort but were not included in multivariate regression models.

To analyze survival outcomes, the Kaplan–Meier method was used to generate survival curves for disease-free survival (DFS) and overall survival (OS), with comparisons made using the log-rank test; as well, a multivariate Cox proportional hazards regression analysis was conducted using a backward stepwise (likelihood ratio) method. Variables included in the model were the pT and pN classifications, as well as the global TNM stage according to both the 7th and 8th editions of the AJCC staging system. Entry and removal criteria were set at *p* = 0.05 and *p* = 0.10, respectively.

The proportional hazards assumption was evaluated through log-minus-log plots and hazard ratios (HR) with 95% confidence intervals (CIs) reported to estimate the relative risk of recurrence and death associated with each variable. To explore the potential impact of adjuvant therapy on survival outcomes, a subgroup analysis was also conducted by stratifying patients according to whether they received postoperative treatment.

Finally, a systematic literature review was conducted to contextualize the study findings, comparing results with previously published research on TNM staging and pancreatic cancer prognosis.

## 3. Results

### 3.1. Patient and Tumor Characteristic

A total of 214 patients with a confirmed histopathological diagnosis of pancreatic adenocarcinoma were included in the study. The clinicopathological characteristics of these patients are summarized in Table 3.

A slight female predominance was observed in the cohort, with women comprising 53.27% and men 46.73% of the study population. The average age at diagnosis was 68.53 years for male patients (range: 44–83 years) and 70.27 years for female patients (range: 38–86 years), suggesting that women were, on average, slightly older at the time of diagnosis.

The anatomical distribution of tumors demonstrated variability across patients; however, the pancreas constituted the predominant site of origin, with 87.38% of cases arising from this organ. A smaller proportion of tumors, 11.68%, originated from the common bile duct and, in 0.94% of cases, the precise location of the tumor could not be definitively determined. For the aim of the present study ampullary tumors were not included.

The size of the tumors also showed considerable variability within the cohort. The mean tumor size was 29.11 mm, with individual tumor measurements ranging from as small as 2 mm to as large as 80 mm. This variation underscores the heterogeneity of tumor presentation, highlighting the importance of staging systems that allow the best categorization of tumors based on size and extent.

To assess the potential prognostic significance, the histological grade of the tumors was reviewed. Tumors were classified into three main categories based on their differentiation: 39.72% were well-differentiated, indicating a more organized cellular architecture and potentially less aggressive behavior; 44.86% were moderately differentiated, representing an intermediate grade with variable biological behavior; and 14.49% were classified as poorly differentiated, typically associated with a more aggressive course and higher metastatic potential. Additionally, in 0.93% of cases, histological grading data were unavailable, limiting the ability to assess differentiation status for these tumors. For analytical purposes, tumors were subsequently grouped into low-grade (39.72%) and high-grade (59.35%) categories, where the high-grade group encompassed both moderately and poorly differentiated tumors, along with 0.93% of cases with missing information.

The presence of lymphovascular invasion, an indicator of the tumor’s ability to spread through the lymphatic and vascular systems, was reported in 41.59% of cases. In contrast, an absence of lymphovascular invasion was reported in 55.61% of cases. In 2.8% of the cohort, however, this parameter was not explicitly documented, leaving uncertainty regarding its presence or absence.

Similarly, perineural invasion, a marker of tumor aggressiveness and potential for local recurrence, was detected in 52.34% of cases. In 15.89% of patients, there was no evidence of perineural invasion, whereas, in 31.78% of cases, the pathology report did not specify the presence of this feature, introducing a limitation in data completeness.

Lymph node status was evaluated to assess the extent of regional spread of the disease. The median number of lymph nodes examined was 13 (range 0–53). Based on this, patients were categorized into two groups: those with fewer than 13 nodes retrieved (n = 102; 47.7%) and those with 13 or more nodes retrieved (n = 109; 50.9%). Concerning the number of positive lymph nodes, the median number of positive nodes was 1 (range 0–17). Similarly, patients were divided into two groups according to nodal involvement: those with no positive nodes (n = 100; 46.73%) and those with one or more positive nodes (n = 111; 51.87%). However, in 1.4% of patients (n = 3), lymph nodes were not included in the analysis, preventing a determination of nodal status in these cases.

Among the 214 patients included in the study, 7 patients (3.3%) received neoadjuvant therapy prior to surgery, while the vast majority—207 patients (96.7%)—did not undergo any neoadjuvant treatment. On the other hand, 96 patients (44.86%) received adjuvant therapy following surgical resection, while 108 patients (50.47%) did not receive any adjuvant treatment. In 10 cases (4.67%), information regarding the administration of adjuvant therapy was not available in the medical records.

The staging of patients was performed according to the 7th and 8th editions of the AJCC TNM classification system to determine potential discrepancies between editions and their impact on disease categorization.

Using the 7th edition, the distribution of patients across TNM stages was as follows: 10.28% were categorized as stage IA, 21.03% as stage IB, 13.55% as stage IIA, and 51.40% as stage IIB. In 3.74% of cases, staging could not be determined due to incomplete data.

After reclassifying the cases using the 8th edition, notable changes were observed: 13.08% of cases were reclassified as stage IA, 18.22% as stage IB, 7.01% as stage IIA, 37.38% as stage IIB, and 14.02% as stage III. In 10.28% of cases, staging could not be assigned due to missing information.

These results illustrate how modifications in tumor size thresholds and lymph node classification criteria in the 8th edition influenced patient distribution across stages. The most striking changes were observed in the stage II category, where a significant decrease in the number of stage IIA and IIB cases was noted in the 8th edition, while the proportion of stage III cases increased substantially.

To further investigate disease progression, an analysis of tumor development was conducted. The median time from surgery to disease recurrence was 12 months, with an interquartile range (IQR) from 5 months (25th percentile) to 21 months (75th percentile). In 37.38% of patients, there was no evidence of disease progression during follow-up. However, 57.94% of patients experienced disease progression, emphasizing the aggressive nature of pancreatic adenocarcinoma. In 4.67% of cases, progression status remained unknown due to insufficient follow-up data.

Regarding overall survival, the median follow-up time until death was 20 months, with an interquartile range of 7 months (25th percentile) to 36 months (75th percentile). The majority of patients succumbed to the disease during the study period. Mortality was recorded in 62.15% of cases, whereas 34.11% of patients remained alive at the last follow-up. In 3.74% of cases, survival status was not documented, either due to the loss of follow-up or insufficient medical records.

These findings underscore the high mortality rate associated with pancreatic adenocarcinoma and highlight the importance of prognostic factors such as lymph node involvement, TNM staging, and tumor differentiation in predicting patient outcomes.

### 3.2. Association Between TNM Classifications and Clinical Outcomes

The transition from the 7th to the 8th edition of the TNM classification system introduced substantial changes in the distribution of cases across pT, pN, and overall TNM categories. These modifications had a notable impact on patient stratification and the clinical relevance of staging.

A significant redistribution of cases was observed in the pT classification, as changes in tumor size thresholds in the 8th edition led to reclassification across multiple categories. The association between the 7th and 8th editions was statistically significant (χ^2^(4) = 13.015, *p* = 0.011), reflecting the non-equivalence between editions and suggesting that the revised tumor size criteria better distinguish tumor progression stages.

In terms of nodal classification (pN), a strong association was found between the two editions (*p* < 0.001). The incorporation of the N2 category (≥4 positive lymph nodes) in the 8th edition provided greater granularity, enabling a more accurate stratification of lymph node involvement and capturing distinctions that were not previously differentiated.

The overall TNM stage also demonstrated significant shifts between editions (*p* < 0.001), with the most prominent reclassifications occurring in stage IIB and stage III. These changes underscore the combined effect of the revised tumor and nodal criteria on staging, resulting in a more nuanced classification of disease severity.

When examining clinical outcomes, TNM staging from the 7th edition was significantly associated with disease progression (*p* = 0.008), with higher stages correlating with increased recurrence. However, no significant association was found between TNM stage and mortality in the 7th edition (*p* = 0.117), suggesting that, while the 7th edition could predict recurrence, it may be limited in predicting overall survival.

Similarly, in the 8th edition, TNM stage remained significantly associated with disease progression (*p* = 0.011), confirming that the updated system retains its prognostic value for recurrence. In contrast, no significant association was observed between TNM stage and mortality (*p* = 0.215), aligning with findings from the 7th edition and indicating that TNM stage, in either version, may not independently predict patient survival in this cohort.

Overall, the 8th edition of the TNM system enhanced the stratification of both tumor size and lymph node involvement, improving the classification of disease progression. However, neither edition showed a statistically significant relationship between TNM stage and patient mortality, highlighting a potential limitation of TNM staging when used alone as a prognostic tool for survival.

### 3.3. Disease-Free and Overall Survival According to TNM

The comparison between the 7th and 8th editions of the AJCC TNM staging system revealed notable differences in their ability to stratify patients with pancreatic adenocarcinoma based on disease-free survival (DFS) and overall survival (OS).

**Figure 1:**
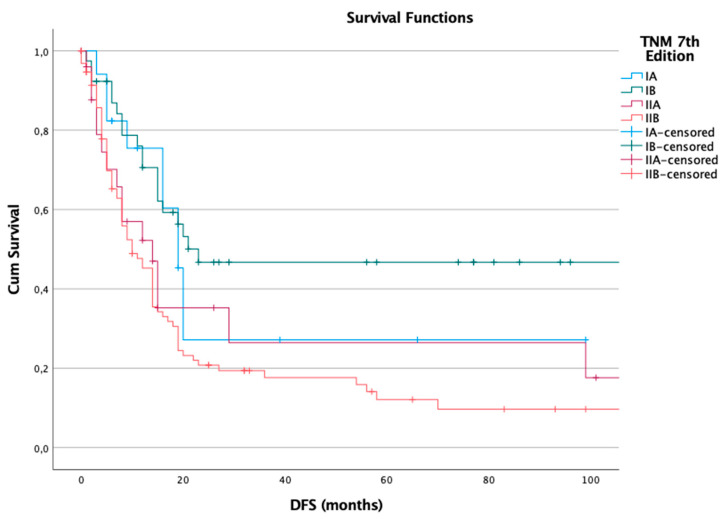
Disease-free survival (DFS) in the 7th edition.

For disease-free survival, the 7th edition demonstrated a clear and consistent decline in median DFS as the stage increased, suggesting a good prognostic performance in predicting the risk of recurrence. Among the 178 patients evaluated, 120 (67%) experienced recurrence during follow-up. The median DFS for each stage was as follows: 19 months for stage IA, 23 months for stage IB, 14 months for stage IIA, and 10 months for stage IIB. The differences observed were statistically significant, as confirmed by the log-rank test (*p* = 0.002), indicating that the 7th edition was effective in differentiating recurrence risk among the stages (Figure 1).

To further clarify patient attrition over time, the number of individuals remaining at risk at different follow-up intervals is provided in supplementary tables, which accompany the Kaplan–Meier survival curves for both DFS and OS (Table 4, Table 5, Table 6 and Table 7). This additional data helps to contextualize the survival curves and supports the interpretation of time-to-event dynamics in each TNM category.

**Figure 2:**
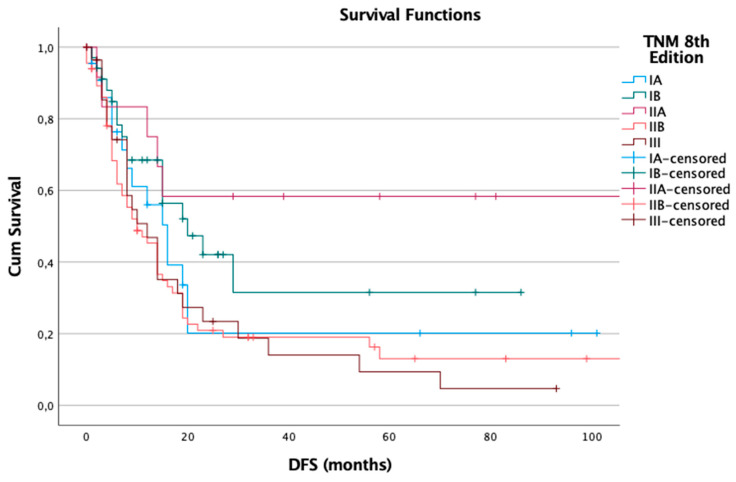
Disease-free survival (DFS) in the 8th edition.

A similar trend was observed when applying the 8th edition of the TNM system, which included 166 patients, of whom 113 (68%) experienced recurrence. The median DFS values for this cohort were 16 months for stage IA, 20 months for stage IB, 129 months for stage IIA (a value that likely reflects censored data rather than an actual survival estimate), 10 months for stage IIB, and 12 months for stage III. Again, the differences among stages were statistically significant (log-rank *p* = 0.023), supporting the revised edition’s ability to distinguish recurrence risk despite the presence of potentially skewed values due to censored cases (Figure 2).

**Figure 3:**
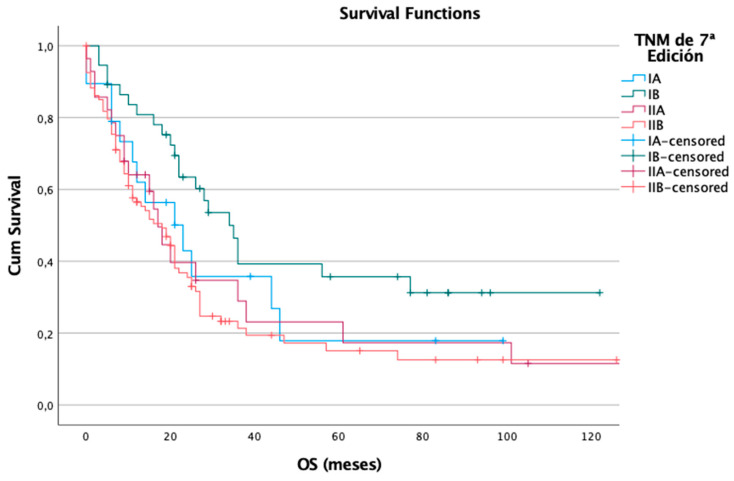
Overall survival in the 7th edition.

In terms of overall survival, the 7th edition also exhibited a stage-dependent pattern, aligning with its observed utility in DFS stratification. Out of the 178 patients assessed, 127 (71%) died during the follow-up period. Median OS decreased with increasing stage: 23 months for stage IA, 34 months for stage IB, 17 months for stage IIA, and 18 months for stage IIB. These findings were statistically significant according to the log-rank test (*p* = 0.028), indicating that the 7th edition was able to effectively differentiate mortality risk across stages (Figure 3).

**Figure 4:**
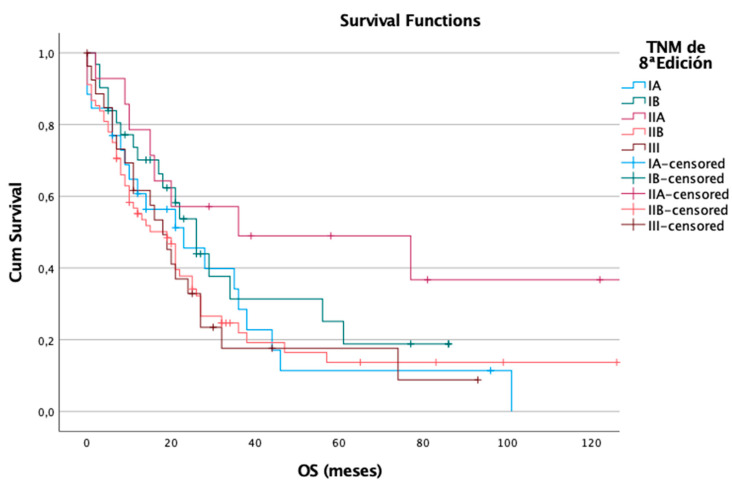
Overall survival in the 8th edition.

However, when OS was analyzed under the 8th edition, the staging system appeared to show less distinct separation between groups. Among the 166 patients analyzed, 120 (72%) died. The median OS was 23 months for stage IA, 26 months for stage IB, 36 months for stage IIA, 19 months for stage IIB, and 18 months for stage III. Although there were numerical differences, particularly the unexpectedly high OS in stage IIA (likely influenced by censored observations), these differences did not reach statistical significance (log-rank *p* = 0.144). This suggests that, in contrast to its performance in predicting recurrence, the 8th edition may have a reduced ability to distinguish between stages in terms of overall survival outcomes (Figure 4).

In the multivariate Cox regression analysis, several variables remained independently associated with recurrence (Table 8). For DFS, only the 7th edition TNM staging system remained in the final model. Although TNM7 stage I showed a trend toward reduced recurrence risk (HR = 0.472; 95% CI: 0.198–1.123; *p* = 0.090), this association did not reach statistical significance. All other variables, including T and N categories from both editions, were excluded in the final steps of model selection. In the final multivariate model for overall survival, TNM staging from the 7th edition was the only significant predictor. Patients classified in TNM7 stage I had a significantly lower risk of death (HR = 0.398; 95% CI: 0.182–0.871; *p* = 0.021), confirming its independent prognostic value. Other categories did not show significant associations, and variables from the 8th edition were excluded during model refinement.

In the final multivariable Cox regression model stratified by adjuvant treatment (Table 9), both pathological T category (pT) and the 8th edition TNM staging system remained significant prognostic factors for disease-free survival. Specifically, pT categories from both the 7th and 8th editions were independently associated with an increased risk of recurrence. For example, pT1 from the 8th edition was associated with a hazard ratio of 2.458 (95% CI: 1.286–4.698; *p* = 0.006), while pT2 showed a non-significant trend (HR = 2.109, 95% CI: 0.865–5.143; *p* = 0.101).

In contrast, the 8th edition TNM stage categories demonstrated a significant protective effect. TNM stage 1 was associated with an HR of 0.187 (95% CI: 0.067–0.520; *p* = 0.001), and TNM stage 2 with an HR of 0.177 (95% CI: 0.046–0.671; *p* = 0.011). The nodal status from the 7th edition (pN) also retained independent prognostic value (HR = 0.318, 95% CI: 0.120–0.843; *p* = 0.021), indicating a reduced risk of recurrence.

In the final Cox model, based on overall survival and incorporating both pathological and staging variables, the 7th edition TNM stage (particularly TNM stage 1) emerged as the only variable independently associated with improved survival outcomes. TNM stage 1 had a hazard ratio of 0.376 (95% CI: 0.162–0.874; *p* = 0.023), confirming its protective role. Other covariates, including pT and pN from both editions, did not retain statistical significance in the fully adjusted model. (The full stepwise Cox regression process for stratified groups, including intermediate models and covariate selection, is provided in the Supplementary Material).

## 4. Discussion

The results of this study reveal notable differences in pT classification between the 7th and 8th editions, particularly in the transition from T1 to T2 categories, where several cases were reclassified under the new criteria. This redistribution suggests that changes in the assignment criteria were made in the updated edition, which may have implications for clinical interpretation, particularly when comparing historical data with current classifications.

Considering that tumor size is a fundamental element in pancreatic cancer staging, its modification in the 8th edition may influence treatment planning and prognostic assessment. The standardization of size-based cutoffs in T classification seeks to reduce variability among pathologists and improve the consistency of staging across institutions. However, these changes also introduce challenges in comparing past studies, as tumors that were previously classified under one category in the 7th edition may now be placed into a different category in the 8th edition, affecting longitudinal analyses.

In terms of pN classification, the results indicate that changes in lymph node staging were not random, but rather reflect methodological modifications aimed at improving case stratification. The introduction of N2 (≥4 positive nodes) in the 8th edition allowed for a more differentiated classification of nodal involvement, recognizing that a higher nodal burden is associated with worse prognosis. This reorganization could have important clinical implications, particularly in prognostic assessment and therapeutic decision-making, as it provides a clearer distinction between cases with limited nodal spread (N1: 1–3 positive nodes) and those with extensive nodal disease (N2: ≥4 positive nodes).

Although the TNM classification between editions showed statistically significant variation, it is important to acknowledge that, in this analysis, a few cells had expected values below 5, suggesting that some groups had a small sample size. This could affect the robustness of the Chi-squared test, as low frequencies in contingency tables may introduce variability in statistical outcomes. However, despite this limitation, the high statistical significance of the findings suggests that the observed differences are unlikely to be due to chance, but instead reflect structural changes introduced in the 8th edition, leading to the systematic re-classification of cases.

An analysis of the contingency table results shows that disease recurrence was more frequent in patients classified as stage IIB in the 7th edition, where 71.15% of cases (74 out of 104) presented recurrence. In contrast, early-stage tumors (stages IA and IB) exhibited significantly lower recurrence rates. These findings suggest that the 7th edition TNM classification had a strong predictive value for disease recurrence, which is clinically relevant for patient management, as staging is one of the key factors guiding follow-up strategies and treatment decisions.

When evaluating the association between the 7th edition TNM classification and mortality, the data show higher mortality rates in patients with advanced-stage tumors (stage IIB: 71 deaths out of 105 cases). The lack of statistical significance observed in this analysis suggests that additional variables, independent of TNM staging, may be impacting mortality outcomes. It is possible that factors such as comorbidities and individual biological characteristics play a key role in determining patient clinical course. The lack of a significant association between TNM classification and mortality underscores the multifactorial nature of survival in pancreatic cancer and points to the need for incorporating additional prognostic markers.

In the 8th edition, advanced stages (IIB and III) continued to show higher recurrence rates, with 51 out of 75 stage IIB patients and 24 out of 29 stage III patients developing disease recurrence. As expected, recurrence rates were lower in earlier stages (IA and IB). These results reinforce the utility of TNM staging in predicting tumor recurrence and suggest that the modifications introduced in the 8th edition maintained the discriminatory capacity of the classification system in identifying patients at higher risk of recurrence or disease progression.

As observed in the 7th edition, the data suggest that mortality rates were higher in advanced stages in the 8th edition (stage IIB: 51 deaths out of 75 cases, and stage III: 21 deaths out of 30 cases). However, the absence of statistical significance in this association once more suggests that other factors may be influencing clinical outcomes.

The results of our survival analysis confirm that the AJCC TNM staging system, in both its 7th and 8th editions, offers valuable prognostic information regarding tumor progression in patients with pancreatic adenocarcinoma. Both versions showed a progressive decrease in disease-free survival (DFS) with increasing stage, and the differences were statistically significant, supporting the use of TNM staging for risk stratification following surgery.

Nevertheless, when analyzing overall survival (OS), some differences emerged. While the 7th edition significantly distinguished between stages with different mortality risks, the 8th edition did not achieve statistical significance, despite a similar trend in survival medians. This discrepancy may reflect the increasing complexity of factors influencing long-term survival beyond pathological staging alone. The redistribution of patients between stages in the newer system (which improves proportionality but may dilute clinical differences between subgroups), individualized follow-up protocols, and patient-specific factors (e.g., age, comorbidities, performance status) likely play a larger role in determining OS. This difference is particularly notable in stage IIB, which in both editions included a large proportion of patients and showed the worst outcomes in terms of both recurrence and mortality. This consistency reinforces the clinical relevance of stage IIB as a high-risk group.

These univariate findings were further supported by multivariate analysis using Cox proportional hazards regression models, and the findings highlight a critical distinction in the prognostic performance of the 7th and 8th editions of the AJCC TNM staging system for pancreatic adenocarcinoma. While the 8th edition introduced refined criteria based on tumor size and nodal burden, its variables did not remain in the final multivariate models, suggesting limited independent prognostic value for both recurrence and survival when adjusted for other factors.

Conversely, the 7th edition TNM classification showed stronger performance in both endpoints. Although its association with disease-free recurrence did not reach significance, a clear trend was observed. Most notably, TNM7 stage I was an independent predictor of overall survival, underscoring the robustness of the 7th edition in capturing mortality-related risk.

These results suggest that, despite the anatomical improvements in the 8th edition, the 7th edition may still better reflect biological behavior and long-term outcomes, particularly when used alone without integrating additional clinical or molecular markers. The findings support the idea that TNM staging, while essential, may require complementary factors to enhance prognostic precision, especially in the context of modern treatment strategies.

In the final multivariable Cox regression model stratified by adjuvant treatment, the final models highlight distinct prognostic capacities of the TNM staging systems and pathological classifications, depending on the outcome assessed. For disease-free survival, both the 7th and 8th edition pT categories consistently correlated with increased risk, while the 8th edition TNM staging appeared more robust in identifying patients at lower recurrence risk. This suggests an improved discriminatory ability of the 8th edition staging for predicting relapse, aligning with the recent literature that supports its refinement over prior versions.

Conversely, for overall survival, the 7th edition TNM classification—specifically, stage I—demonstrated the strongest and most consistent association with better outcomes, particularly in patients who received adjuvant therapy. The diminished significance of pT and nodal status in the final OS model may reflect the influence of systemic therapy or other clinical factors that modulate long-term survival beyond anatomical extent alone.

Taken together, these findings underscore the complementary prognostic roles of both editions: the 8th edition may better stratify early recurrence risk (DFS), whereas the 7th edition retains relevance for long-term survival (OS), especially in treated populations. These observations support a nuanced clinical use of staging systems, depending on the outcome of interest.

Despite the strengths of this study, several limitations must be acknowledged. First, while the study aimed to include all patients diagnosed with pancreatic adenocarcinoma between 2000 and 2022, the distribution of cases was not uniform across time periods. A significant proportion of patients were diagnosed between 2008 and 2015 (102 cases), whereas the earlier period from 2000 to 2007 (52 cases) and the more recent period from 2016 to 2022 (60 cases) had fewer patients. This uneven distribution could introduce selection bias and may limit the generalizability of findings, during which notable advances in surgical techniques, perioperative care, imaging modalities, and adjuvant therapy regimens have occurred. These temporal variations between subgroups could potentially introduce heterogeneity in treatment outcomes and limit the comparability within the cohort. However, since our analysis primarily focuses on pathological staging parameters (tumor size, nodal involvement) that are less likely to be influenced by evolving clinical practices, we believe that the core objectives of this study remain valid. Second, the study did not evaluate the use of adjuvant therapies over time, which is recognized as a limitation. While adjuvant treatments are commonly administered following surgical resection, their impact on long-term survival in pancreatic cancer remains a subject of debate. The exclusion of adjuvant therapy data prevents an assessment of its influence on disease recurrence and overall survival, which could be relevant for understanding treatment-related prognostic differences. However, it is noteworthy that the benefits of adjuvant therapy in pancreatic cancer remain somewhat limited, and studies have demonstrated improvements in survival outcomes in both node-negative and node-positive patients, regardless of adjuvant treatment administration. Future research incorporating detailed treatment data could provide a more comprehensive analysis of the therapeutic impact on disease recurrence and survival. Finally, a significant loss to follow-up was observed in the study, which may have undermined the statistical power of some analyses. Incomplete follow-up data can result in bias in survival estimates, particularly if the patients lost to follow-up had different clinical characteristics compared to those who remained in the study. This limitation highlights the importance of structured follow-up protocols in prospective studies to minimize data loss and ensure a more robust assessment of long-term outcomes.

The 7th edition AJCC staging system for pancreatic adenocarcinoma has been subject to several criticisms, particularly regarding the definitions of T and N classification [1,6,8,14]. One of the main concerns has been the inclusion of “extension beyond the pancreas” as a defining criterion for T3 tumors. Due to the thin structure of the pancreas, a large proportion of tumors inevitably exhibit some degree of extension to the surface, leading to an overclassification of tumors as T3, regardless of their actual size. In some studies, this criterion resulted in up to 90% of resected patients being categorized as T3 [6,14]. A further limitation of the T3 definition in the 7th edition is that the pancreas lacks a true capsule, and its soft tissue surface often contains deep invaginations between the lobes. Additionally, chronic pancreatitis associated with invasive carcinoma may obliterate the boundary between the pancreatic parenchyma and extrapancreatic soft tissue, making the interpretation of “extension beyond the pancreas” highly dependent on individual pathologists’ criteria [1,6,8,13]. This lack of reproducibility raised concerns about staging accuracy, resulting in the 8th edition modifications. Given these limitations, tumor diameter has been proposed as a more objective and reproducible criterion for defining T stage, as it has been recognized as a strong predictor of survival in multiple malignancies, including pancreatic adenocarcinoma [6,15]. The updated size-based cutoffs (T1 ≤2 cm, T2 >2–≤4 cm, T3 >4 cm) align with the criteria used for other gastrointestinal and pancreatic tumors, including pancreatic neuroendocrine tumors [6,15]. Allen et al. conducted an extensive analysis in 1551 patients who had undergone R0 resection, identifying tumor size cutoffs of <2.2 cm and ≥4.8 cm, which were significantly associated with survival and supported the proposed modifications [6]. This evidence reinforces that the size-based staging system is statistically robust and reproducible across institutions. Additionally, Saka et al. found that the median survival time and 3-year survival rates using the new size-based classification were 38, 18, and 13 months, with corresponding survival rates of 52%, 28%, and 10%, respectively (*p* < 0.001) in node-negative pancreatic cancer patients [14]. Furthermore, Schlitter et al. demonstrated that survival discrimination between T2 and T3 pancreatic cancer improved with the 8th edition classification (median survival: T2, 12.7 months; T3, 8.9 months), whereas the 7th edition failed to show significant survival differences (T2, 14.4 months; T3, 12.3 months) [15]. Despite the improvements in T staging, Liu et al. raised an important consideration regarding the prognostic equivalence of tumor size and nodal involvement. Their study found that patients with tumors >4 cm (T3N0M0) had a similar prognosis to those with 1–3 positive nodes (T1–3N1M0) in both the SEER cohort (stage I as reference; stage IIA HR: 1.82; stage IIB HR: 1.88) and institutional series (stage I as reference; stage IIA HR: 1.72; stage IIB HR: 1.70) [1]. These findings suggest that nodal involvement remains a critical factor in prognostic assessment, complementing tumor size-based classification.

Similarly to tumor size, nodal status has been widely recognized as a key prognostic factor in pancreatic cancer, with higher nodal involvement correlating with shorter survival times [1,16,17,18,19,20,21,22,23,24]. The 7th edition AJCC staging system categorized lymph node involvement as either N0 (node-negative) or N1 (node-positive), without further stratification. This binary classification failed to differentiate between patients with limited nodal spread and those with extensive lymph node metastases, leading to heterogeneous survival outcomes within the same stage [6,24]. Most studies have suggested that the number of positive LNs may improve prognostication over the binary designation of negative vs. positive LN involvement so, Morales-Oyarvide et al. confirmed the additional prognostic benefit to this approach, with median OS times of 35.1, 20.6, and 16.8 months in the entire study population for patients with N0, N1, and N2 disease, respectively [24]. Allen et al. evaluated various nodal cutoffs and noted that other gastrointestinal cancers, such as colorectal cancer, classify nodal involvement as N0 (no nodes affected), N1 (1–3 positive nodes), and N2 (≥4 positive nodes) [6]. Their analysis confirmed that the same structure was appropriate for pancreatic cancer, identifying optimal cutoffs of >0.5 positive nodes and ≥3.5 positive nodes [6]. Similarly, Basturk et al. examined nodal metastasis in pancreatic adenocarcinoma and found that 70% of cases had lymph node involvement, with a mean of 18 nodes examined and 3 positive nodes [25]. Their findings aligned with the SEER database, which reported an average of seven lymph nodes examined, with one positive node [16,17,18]. The study confirmed that N-negative patients had a median survival of 35 months, whereas N-positive patients had a median survival of 19 months, reinforcing the prognostic implications of nodal metastasis [19,20,21,22,26]. However, not all studies showed such similar results regarding the prognostic ability of lymph node classification, as Shin et al. validated the 8th edition staging system using data from Korean patients and found no significant difference in overall survival (OS) between pN1 and pN2 (18.1 months vs. 16.9 months; *p* = 0.10) [27].

Allen et al. analyzed the proportional distribution of TNM stages and found that, when applying the 7th edition, 87% of patients were classified as stage II, with only 12% as stage I and 0% as stage III [6]. However, with the 8th edition, staging shifted significantly, with 26% now classified as stage III. A similar trend was observed in the SEER database, where the proportion of stage III cases increased (18.2% vs. 11.7%), while stages I and II cases decreased (23.8% vs. 30.4%). This redistribution suggests that the 8th edition provides a more accurate reflection of disease burden, improving staging proportionality and prognostic precision [6]. Rossell et al., observed that the eighth edition of the TNM staging system provided a more balanced distribution of patients across stages and showed improved prognostic accuracy compared to the seventh edition of the AJCC system. It also allowed for more effective patient reclassification. However, the revised T stage did not show a significant association with survival in either univariate or multivariate analyses, whereas the updated N stage demonstrated a clear ability to discriminate survival outcomes [28].

A notable modification was the redefinition of stage III disease, which now includes both locally advanced tumors (T4N0M0) and tumors with a high lymph node burden (any T, N2, M0) [6]. This change aligns pancreatic cancer staging with other malignancies and may improve treatment stratification for patients with significant nodal involvement [29]. However, some studies proposed that patients with N2 and T4, which have been integrated into stage III and showed different treatment modalities and prognoses, should been divided into IIIA (T1–T3N2M0) and IIIB (T4NAnyM0) [3,13]. Finally, although the new edition of the AJCC has improved the prognostic capacity of pancreatic cancer, there is a need to study other clinical factors to determine the prognosis of this entity [4,30]. Zhong R. et al. created a nomogram with 12 prognostic factors (age, sex, histologic, marital, grade, TNM stage, surgery, extent of lymph node dissection, LNR, tumor size, radiation therapy, and chemotherapy) that included more clinical information to determine the prognosis of patients more accurately and guide their future treatment strategy. In their study surgery, TNM stage and grade exhibited the strongest impact on prognosis among all factors, age and tumor size had a moderate influence on prognosis, and sex and marital status only had a minor effect [4]. Hu et al. concluded that CA19-9 is a robust preoperative prognostic indicator for resected patients. They identified 500 U/mL as the preoperative CA19-9 cutoff point in patients with resected PDAC, and, in their univariate and multivariate analyses, they demonstrated that CA19-9 was the strongest prognostic indicator among all preoperative factors evaluated. They also observed prognostic heterogeneity within each AJCC 8th edition stage when stratified by this cutoff, except for stage III. Furthermore, they developed a modified staging system that incorporated the preoperative CA19-9 value, which showed superior accuracy in predicting patient survival and may assist in selecting the most appropriate candidates for neoadjuvant therapy [5].

## 5. Conclusions

In conclusion, this study provides a comprehensive comparison between the 7th and 8th editions of the AJCC TNM staging system for pancreatic adenocarcinoma, analyzing their performance in stratifying patients according to disease-free survival (DFS) and overall survival (OS) in a real-world clinical setting. Our findings highlight several key observations:

- The 8th edition, which redefines T staging based on tumor size and introduces an N2 category for extensive nodal involvement, demonstrates improved stratification for disease recurrence. In particular, the 8th edition TNM stage showed a strong association with DFS in both univariate and multivariate analyses, supporting its value as a predictor of early relapse risk;
- On the other hand, the 7th edition TNM staging system retained superior prognostic accuracy for overall survival, especially in patients receiving adjuvant therapy. TNM stage I from the 7th edition consistently emerged as an independent protective factor for mortality in multivariate Cox models, whereas variables from the 8th edition did not remain statistically significant;
- These findings underscore the complementary prognostic strengths of both editions: the 8th edition offers enhanced precision for early recurrence risk assessment, while the 7th edition remains relevant for long-term survival prediction;
- The modifications introduced in the 8th edition led to a more balanced distribution of cases across stages, particularly by reducing the overrepresentation of stage IIB and recognizing high nodal burden as stage III. However, these structural improvements did not fully translate into improved OS discrimination, suggesting that long-term outcomes may depend on factors beyond anatomical staging;
- Our results reaffirm the importance of considering additional clinical, pathological, and molecular variables alongside TNM staging to improve prognostic accuracy in pancreatic cancer. Factors such as tumor differentiation, lymphovascular and perineural invasion, preoperative CA19-9 levels, and treatment variables should be integrated into future risk models;
- Finally, although both TNM editions provide valuable information, their optimal use may vary depending on the clinical objective: the 8th edition is better suited for stratifying recurrence risk, while the 7th edition offers stronger predictive value for survival. Future research should aim to integrate staging systems with dynamic, multifactorial tools—such as nomograms—to support personalized decision-making in pancreatic cancer management.

## Figures and Tables

**Table 1:** The 7th and 8th edition AJCC staging definitions for pancreatic adenocarcinoma.

7th Edition		8th Edition	
T1	Confined to the pancreas, ≤2 cm in greatest dimension	T1	Confined to the pancreas, ≤2 cm in greatest dimension
T2	Confined to the pancreas, >2 cm in greatest dimension	T2	Confined to the pancreas, >2 cm and ≤4 cm in greatest dimension
T3	Extends beyond the pancreas but without involvement of the celiac axis or superior mesenteric artery	T3	Confined to the pancreas, >4 cm in greatest dimension
T4	Involvement of the celiac axis or superior mesenteric artery (unresectable tumor)	T4	Tumor involves the celiac axis and/or superior mesenteric artery and/or common hepatic artery, regardless of size
N0	No regional lymph node metastasis	N0	No regional lymph node metastasis
N1	Regional lymph node metastasis	N1	Metastasis in 1 to 3 regional lymph nodes
		N2	Metastasis in 4 or more regional lymph nodes
M0	No distant metastasis	M0	No distant metastasis
M1	Distant metastasis	M1	Distant metastasis

**Table 2:** AJCC pathologic stage groupings (7th and 8th edition) for pancreatic adenocarcinoma according to TNM.

7th Edition				8th Edition			
Stage	T	N	M	Stage	T	N	M
IA	T1	N0	M0	IA	T1	N0	M0
IB	T2	N0	M0	IB	T2	N0	M0
IIA	T3	N0	M0	IIA	T3	N0	M0
IIB	T1–T3	N1	M0	IIB	T1–T3	N1	M0
III	T4	Any N	M0	III	Any T	N2	M0
					T4	Any N	
IV	Any T	Any N	M1	IV	Any T	Any N	M1

**Table 3:** Clinicopathological characteristics of patients undergoing pancreatic resection included in the study and staging according to the 8th and 7th AJCC editions.

Clinical Characteristics	n	%	Clinical Characteristics	n	%
A ge			Perineural Invasion		
<65 years	67	30. 99%	No	34	15.89%
>65 years	147	69.01%	Yes	112	52.34%
Sex			N/A	68	31.78%
Male	100	46.73%	Lymph Node Involvement		
Female	114	53.27%	N/A	3	1.4%
Tumor Location			No	100	46.73%
Pancreas	187	87.38%	Yes	111	51.87%
Common bile duct	25	11.68%	Tumor Recurrence		
N/A	2	0.94%	No	80	37.38%
Histological Grade			Yes	124	57.94%
High	127	59.35%	N/A	10	4.67%
Low	85	39.72%	Death		
N/A	2	0.93%			
Lymphovascular Invasion			No	73	34.11%
No	119	55.61%	Yes	133	62.15%
Yes	89	41.59%	N/A	8	3.74%
N/A	6	2.8%			
Neoadjuvant Therapy			Adjuvant Therapy		
No	207	96.7%	No	108	50.47%
Yes	7	3.3%	Yes	96	44.86%
N/A	0	0%	N/A	10	4.67%
AJCC 8th Edition Stage Distribution					
IA	28	13.08%	III	30	14.02%
IB	39	18.22%	IV	0	0%
IIA	15	7.01%	N/A	22	10.28%
IIB	80	37.38%			
AJCC 7th Edition Stage Distribution					
IA	22	10.28%	III	0	0%
IB	45	21.03%	IV	0	0%
IIA	29	13.55%	N/A	8	3.74%
IIB	110	51.40%			

**Table 4:** Number of at-risk disease-free survival (DFS) cases in the 7th edition.

Time (Months)	Stage IA	Stage IB	Stage IIA	Stage IIB
0	18	36	26	98
12	12	25	15	50
24	8	18	10	30
36	5	11	5	15

**Table 5:** Disease-free survival (DFS) in the 8th edition.

Time (Months)	Stage IA	Stage IB	Stage IIA	Stage IIB	Stage III
0	23	33	14	70	26
12	16	22	10	40	12
24	10	16	8	25	7
36	6	10	5	12	3

**Table 6:** Overall survival in the 7th edition.

Time (Months)	Stage IA	Stage IB	Stage IIA	Stage IIB
0	18	36	26	98
12	14	27	18	60
24	9	19	12	35
36	5	12	7	18

**Table 7:** Overall survival in the 8th edition.

Time (Months)	Stage IA	Stage IB	Stage IIA	Stage IIB	Stage III
0	23	33	14	70	26
12	18	25	11	45	15
24	12	19	9	30	9
36	7	11	6	15	4

**Table 8:** Global Cox regression analysis—disease-free and overall survival (final model).

Model DFS			
Variables	HR	95% CI	*p*-value
TNM7 (IA)	Reference	-	0.012
TNM7 (IB)	0.472	0.198–11.123	0.09
TNM7 (IIA)	1.22	0.537–2.772	0.635
TNM7 (IIB)	1.328	0.661–2.666	0.425
Model OS			
Variables	HR	95% CI	*p*-value
TNM7 (IA)	Reference	-	0.45
TNM7 (IB)	0.398	0.182–0.871	0.021
TNM7 (IIA)	0.819	0.393–1.706	0.594
TNM7 (IIB)	0.864	0.455–1.642	0.656

**Table 9:** Stratified by adjuvant treatment Cox regression analysis—disease-free and overall survival (final model).

Model DFS			
V ariables	HR	95% CI	*p*-value
pT7 (T1)	Reference	-	0.02
pT7 (T2)	2. 15	1.128–4.098	0.02
pT7 (T3)	2.481	1.306–4.711	0.006
pT8 (T1)	Reference	-	0.024
pT8 (T2)	2.458	1.286–4.698	0.006
pT8 (T3)	2.109	0.865–5.143	0.101
pN7	0.318	0.120–0.843	0.021
TNM8 (IA)	Reference	-	0.007
TNM8 (IB)	0.187	0.067–0.520	0.001
TNM8 (IIA)	0.177	0.46–0.671	0.011
TNM8 (IIB)	1.535	0.870–2.711	0.139
Model OS			
Variables	HR	95% CI	*p*-value
TNM7 (IA)	Reference	-	0.57
TNM7 (IB)	0.376	0.162–0.874	0.023
TNM7 (IIA)	0.755	0.344–1.659	0.485
TNM7 (IIB)	0.806	0.399–1.629	0.548

## Data Availability

The data presented in this study, although anonymized to avoid violating patient confidentiality, are not publicly available due to ethical restrictions.

## Supplementary Materials

The following supporting information can be downloaded at: https://www.mdpi.com/article/10.3390/cancers17111890/s1.
