# Supplementary material for: Prognostic Validity of the Eighth Edition of the U.S. Joint Committee on Cancer TNM Staging System for Pancreatic Adenocarcinomas: An Analysis of 214 Patients at a Spanish Center
Source: Cancers (Basel). 2025 Jun 5;17(11):1890. doi: 10.3390/cancers17111890 (PMC12153685; doi:10.3390/cancers17111890)
Supplement: Supplementary file 1 [file cancers-17-01890-s001.zip › cancers-3598476-supplementary.pdf]

Global Cox Regression Analysis – Disease-Free Survival[b]

		B	SE	Wald	df	Sig.	Exp(B)	95,0% CI for Exp(B) Lower	Upper
Step 1	pT de 7ª Edición			4,605	2	,100			
	pT de 7ª Edición(1)	,703	,328	4,605	1	,032	2,020	1,063	3,839
	pT de 7ª Edición(2)	,563	,366	2,368	1	,124	1,755	,857	3,594
	pT de 8ª Edición			7,100	2	,029			
	pT de 8ª Edición(1)	,849	,327	6,739	1	,009	2,337	1,231	4,436
	pT de 8ª Edición(2)	,906	,454	3,982	1	,046	2,476	1,016	6,030
	pN de 7ª Edición	-,975	,545	3,197	1	,074	,377	,130	1,098
	pN de 8ª Edición			,705	1[a]	,401			
	pN de 8ª Edición (1)	,231	,275	,705	1	,401	1,260	,735	2,159
	TNM de 7ª Edición			4,274	2[a]	,118			
	TNM de 7ª Edición(1)	-,878	,565	2,417	1	,120	,416	,137	1,257
	TNM de 7ª Edición(2)	-,010	,579	,000	1	,987	,990	,318	3,079
	TNM de 8ªEdición			5,827	2[a]	,054			
	TNM de 8ªEdición(1)	-1,050	,545	3,708	1	,054	,350	,120	1,019
	TNM de 8ªEdición(2)	-1,589	,703	5,109	1	,024	,204	,051	,810
Step 2	pT de 7ª Edición			4,605	2	,100			
	pT de 7ª Edición(1)	,703	,328	4,605	1	,032	2,020	1,063	3,839
	pT de 7ª Edición(2)	,563	,366	2,368	1	,124	1,755	,857	3,594
	pT de 8ª Edición			7,100	2	,029			
	pT de 8ª Edición(1)	,849	,327	6,739	1	,009	2,337	1,231	4,436
	pT de 8ª Edición(2)	,906	,454	3,982	1	,046	2,476	1,016	6,030
	pN de 7ª Edición	-,975	,545	3,197	1	,074	,377	,130	1,098
	TNM de 7ª Edición			4,274	2[a]	,118			
	TNM de 7ª Edición(1)	-,878	,565	2,417	1	,120	,416	,137	1,257
	TNM de 7ª Edición(2)	-,010	,579	,000	1	,987	,990	,318	3,079
	TNM de 8ªEdición			6,073	3[a]	,108			
	TNM de 8ªEdición(1)	-1,050	,545	3,708	1	,054	,350	,120	1,019
	TNM de 8ªEdición(2)	-1,589	,703	5,109	1	,024	,204	,051	,810
	TNM de 8ªEdición(3)	,231	,275	,705	1	,401	1,260	,735	2,159
Step 3	pT de 7ª Edición			4,566	2	,102			
	pT de 7ª Edición(1)	,706	,331	4,557	1	,033	2,025	1,059	3,870
	pT de 7ª Edición(2)	,612	,372	2,704	1	,100	1,845	,889	3,829
	pT de 8ª Edición			3,719	2	,156			
	pT de 8ª Edición(1)	,478	,253	3,567	1	,059	1,612	,982	2,647
	pT de 8ª Edición(2)	,211	,337	,392	1	,531	1,235	,638	2,392
	pN de 7ª Edición	-,390	,445	,769	1	,381	,677	,283	1,620
	TNM de 7ª Edición			8,436	2[a]	,015			
	TNM de 7ª Edición(1)	-1,374	,505	7,409	1	,006	,253	,094	,681
	TNM de 7ª Edición(2)	-,502	,553	,824	1	,364	,605	,205	1,790
Step 4	pT de 7ª Edición			4,566	2	,102			
	pT de 7ª Edición(1)	,706	,331	4,557	1	,033	2,025	1,059	3,870
	pT de 7ª Edición(2)	,612	,372	2,704	1	,100	1,845	,889	3,829
	pT de 8ª Edición			3,719	2	,156			
	pT de 8ª Edición(1)	,478	,253	3,567	1	,059	1,612	,982	2,647
	pT de 8ª Edición(2)	,211	,337	,392	1	,531	1,235	,638	2,392
	TNM de 7ª Edición			10,736	3	,013			
	TNM de 7ª Edición(1)	-1,374	,505	7,409	1	,006	,253	,094	,681
	TNM de 7ª Edición(2)	-,502	,553	,824	1	,364	,605	,205	1,790
	TNM de 7ª Edición(3)	-,390	,445	,769	1	,381	,677	,283	1,620
Step 5	pT de 7ª Edición			4,170	2	,124			
	pT de 7ª Edición(1)	,664	,325	4,158	1	,041	1,942	1,026	3,676
	pT de 7ª Edición(2)	,527	,369	2,048	1	,152	1,695	,823	3,489
	TNM de 7ª Edición			9,011	3	,029			
	TNM de 7ª Edición(1)	-1,039	,466	4,979	1	,026	,354	,142	,881
	TNM de 7ª Edición(2)	-,173	,519	,112	1	,738	,841	,304	2,323
	TNM de 7ª Edición(3)	-,089	,411	,047	1	,828	,915	,408	2,049
Step 6	TNM de 7ª Edición			10,994	3	,012			
	TNM de 7ª Edición(1)	-,751	,442	2,882	1	,090	,472	,198	1,123
	TNM de 7ª Edición(2)	,199	,419	,226	1	,635	1,220	,537	2,772
	TNM de 7ª Edición(3)	,284	,356	,635	1	,425	1,328	,661	2,666

a. Degree of freedom reduced because of constant or linearly dependent covariates

b. Constant or Linearly Dependent Covariates pN de 7ª Edición = TNM de 7ª Edición(3) ;

Global Cox Regression Analysis – Overall Survival[b]

		B	SE	Wald	df	Sig.	Exp(B)	95,0% CI for Exp(B) Lower	Upper
Step 1	pT de 7ª Edición			,990	2	,610			
	pT de 7ª Edición(1)	,273	,297	,845	1	,358	1,314	,734	2,353
	pT de 7ª Edición(2)	,307	,345	,792	1	,373	1,360	,691	2,675
	pT de 8ª Edición			5,526	2	,063			
	pT de 8ª Edición(1)	,770	,328	5,519	1	,019	2,159	1,136	4,105
	pT de 8ª Edición(2)	,566	,468	1,465	1	,226	1,761	,704	4,406
	pN de 7ª Edición	-,945	,517	3,338	1	,068	,389	,141	1,071
	pN de 8ª Edición			,021	1[a]	,885			
	pN de 8ª Edición (1)	,042	,291	,021	1	,885	1,043	,589	1,846
	TNM de 7ª Edición			4,203	2[a]	,122			
	TNM de 7ª Edición(1)	-,925	,476	3,767	1	,052	,397	,156	1,009
	TNM de 7ª Edición(2)	-,354	,513	,475	1	,491	,702	,257	1,920
	TNM de 8ªEdición			3,649	2[a]	,161			
	TNM de 8ªEdición(1)	-,894	,495	3,263	1	,071	,409	,155	1,079
	TNM de 8ªEdición(2)	-,922	,636	2,101	1	,147	,398	,114	1,383
Step 2	pT de 7ª Edición			,990	2	,610			
	pT de 7ª Edición(1)	,273	,297	,845	1	,358	1,314	,734	2,353
	pT de 7ª Edición(2)	,307	,345	,792	1	,373	1,360	,691	2,675
	pT de 8ª Edición			5,526	2	,063			
	pT de 8ª Edición(1)	,770	,328	5,519	1	,019	2,159	1,136	4,105
	pT de 8ª Edición(2)	,566	,468	1,465	1	,226	1,761	,704	4,406
	pN de 7ª Edición	-,945	,517	3,338	1	,068	,389	,141	1,071
	TNM de 7ª Edición			4,203	2[a]	,122			
	TNM de 7ª Edición(1)	-,925	,476	3,767	1	,052	,397	,156	1,009
	TNM de 7ª Edición(2)	-,354	,513	,475	1	,491	,702	,257	1,920
	TNM de 8ªEdición			3,650	3[a]	,302			
	TNM de 8ªEdición(1)	-,894	,495	3,263	1	,071	,409	,155	1,079
	TNM de 8ªEdición(2)	-,922	,636	2,101	1	,147	,398	,114	1,383
	TNM de 8ªEdición(3)	,042	,291	,021	1	,885	1,043	,589	1,846
Step 3	pT de 8ª Edición			4,983	2	,083			
	pT de 8ª Edición(1)	,725	,325	4,978	1	,026	2,064	1,092	3,901
	pT de 8ª Edición(2)	,543	,467	1,353	1	,245	1,721	,689	4,296
	pN de 7ª Edición	-,738	,477	2,396	1	,122	,478	,188	1,217
	TNM de 7ª Edición			4,825	2[a]	,090			
	TNM de 7ª Edición(1)	-,802	,459	3,052	1	,081	,449	,182	1,103
	TNM de 7ª Edición(2)	-,100	,410	,059	1	,808	,905	,405	2,022
	TNM de 8ªEdición			3,779	3[a]	,286			
	TNM de 8ªEdición(1)	-,896	,494	3,281	1	,070	,408	,155	1,076
	TNM de 8ªEdición(2)	-,963	,635	2,300	1	,129	,382	,110	1,325
	TNM de 8ªEdición(3)	,022	,285	,006	1	,938	1,022	,585	1,786
Step 4	pT de 8ª Edición			2,652	2	,266			
	pT de 8ª Edición(1)	,357	,236	2,286	1	,131	1,430	,899	2,272
	pT de 8ª Edición(2)	,064	,307	,044	1	,834	1,066	,584	1,947
	pN de 7ª Edición	-,332	,350	,897	1	,344	,718	,361	1,426
	TNM de 7ª Edición			8,226	2[a]	,016			
	TNM de 7ª Edición(1)	-1,130	,427	6,993	1	,008	,323	,140	,746
	TNM de 7ª Edición(2)	-,354	,393	,812	1	,368	,702	,325	1,516
Step 5	pT de 8ª Edición			2,652	2	,266			
	pT de 8ª Edición(1)	,357	,236	2,286	1	,131	1,430	,899	2,272
	pT de 8ª Edición(2)	,064	,307	,044	1	,834	1,066	,584	1,947
	TNM de 7ª Edición			9,248	3	,026			
	TNM de 7ª Edición(1)	-1,130	,427	6,993	1	,008	,323	,140	,746
	TNM de 7ª Edición(2)	-,354	,393	,812	1	,368	,702	,325	1,516
	TNM de 7ª Edición(3)	-,332	,350	,897	1	,344	,718	,361	1,426
Step 6	TNM de 7ª Edición			8,041	3	,045			
	TNM de 7ª Edición(1)	-,922	,400	5,312	1	,021	,398	,182	,871
	TNM de 7ª Edición(2)	-,200	,374	,285	1	,594	,819	,393	1,706
	TNM de 7ª Edición(3)	-,146	,328	,199	1	,656	,864	,455	1,642

a. Degree of freedom reduced because of constant or linearly dependent covariates

b. Constant or Linearly Dependent Covariates pN de 7ª Edición = TNM de 7ª Edición(3) ;

Stratified by adjuvant treatment - Cox Regression Analysis - Disease-Free Survival[b]

		B	SE	Wald	df	Sig.	Exp(B)	95,0% CI for Exp(B) Lower	Upper
Step 1	pT de 7ª Edición			5,892	2	,053			
	pT de 7ª Edición(1)	,856	,354	5,851	1	,016	2,353	1,176	4,706
	pT de 7ª Edición(2)	,761	,395	3,708	1	,054	2,140	,987	4,643
	pT de 8ª Edición			7,517	2	,023			
	pT de 8ª Edición(1)	,899	,330	7,406	1	,006	2,458	1,286	4,698
	pT de 8ª Edición(2)	,818	,456	3,220	1	,073	2,265	,927	5,534
	pN de 7ª Edición	-1,335	,607	4,838	1	,028	,263	,080	,865
	pN de 8ª Edición			2,526	1[a]	,112			
	pN de 8ª Edición (1)	,460	,290	2,526	1	,112	1,584	,898	2,794
	TNM de 7ª Edición			3,598	2[a]	,165			
	TNM de 7ª Edición(1)	-,859	,614	1,960	1	,162	,424	,127	1,410
	TNM de 7ª Edición(2)	-,024	,620	,001	1	,969	,977	,290	3,292
	TNM de 8ªEdición			6,842	2[a]	,033			
	TNM de 8ªEdición(1)	-1,345	,580	5,387	1	,020	,260	,084	,811
	TNM de 8ªEdición(2)	-1,590	,707	5,052	1	,025	,204	,051	,816
Step 2	pT de 7ª Edición			7,806	2	,020			
	pT de 7ª Edición(1)	,765	,329	5,404	1	,020	2,150	1,128	4,098
	pT de 7ª Edición(2)	,909	,327	7,707	1	,006	2,481	1,306	4,711
	pT de 8ª Edición			7,423	2	,024			
	pT de 8ª Edición(1)	,899	,330	7,407	1	,006	2,458	1,286	4,698
	pT de 8ª Edición(2)	,746	,455	2,694	1	,101	2,109	,865	5,143
	pN de 7ª Edición	-1,146	,497	5,308	1	,021	,318	,120	,843
	pN de 8ª Edición			2,184	1[a]	,139			
	pN de 8ª Edición (1)	,429	,290	2,184	1	,139	1,535	,870	2,711
	TNM de 8ªEdición			11,604	2[a]	,003			
	TNM de 8ªEdición(1)	-1,679	,523	10,291	1	,001	,187	,067	,520
	TNM de 8ªEdición(2)	-1,734	,681	6,488	1	,011	,177	,046	,671
Step 3	pT de 7ª Edición			7,806	2	,020			
	pT de 7ª Edición(1)	,765	,329	5,404	1	,020	2,150	1,128	4,098
	pT de 7ª Edición(2)	,909	,327	7,707	1	,006	2,481	1,306	4,711
	pT de 8ª Edición			7,423	2	,024			
	pT de 8ª Edición(1)	,899	,330	7,407	1	,006	2,458	1,286	4,698
	pT de 8ª Edición(2)	,746	,455	2,694	1	,101	2,109	,865	5,143
	pN de 7ª Edición	-1,146	,497	5,308	1	,021	,318	,120	,843
	TNM de 8ªEdición			12,205	3[a]	,007			
	TNM de 8ªEdición(1)	-1,679	,523	10,291	1	,001	,187	,067	,520
	TNM de 8ªEdición(2)	-1,734	,681	6,488	1	,011	,177	,046	,671
	TNM de 8ªEdición(3)	,429	,290	2,184	1	,139	1,535	,870	2,711

a. Degree of freedom reduced because of constant or linearly dependent covariates

b. Constant or Linearly Dependent Covariates S = Stratum effect. pN de 8ª Edición (1) = TNM de 8ªEdición(3) + S ; pN de 8ª Edición (2) = pN de 7ª Edición - TNM de 8ªEdición(3) + S ; TNM de 7ª Edición(3) = pN de 7ª Edición + S ; TNM de 8ªEdición(4) = pN de 7ª Edición - TNM de 8ªEdición(3) + S ;

Stratified by adjuvant treatment – Cox Regression Analysis – Overall Survival[b]

		B	SE	Wald	df	Sig.	Exp(B)	95,0% CI for Exp(B) Lower	Upper
Step 1	pT de 7ª Edición			,405	2	,817			
	pT de 7ª Edición(1)	,190	,308	,380	1	,538	1,209	,661	2,211
	pT de 7ª Edición(2)	,185	,355	,273	1	,602	1,203	,601	2,411
	pT de 8ª Edición			6,731	2	,035			
	pT de 8ª Edición(1)	,876	,339	6,665	1	,010	2,402	1,235	4,673
	pT de 8ª Edición(2)	,581	,478	1,474	1	,225	1,787	,700	4,563
	pN de 7ª Edición	-,947	,554	2,918	1	,088	,388	,131	1,150
	pN de 8ª Edición			,052	1[a]	,819			
	pN de 8ª Edición (1)	-,070	,307	,052	1	,819	,932	,511	1,700
	TNM de 7ª Edición			3,988	2[a]	,136			
	TNM de 7ª Edición(1)	-,939	,507	3,429	1	,064	,391	,145	1,056
	TNM de 7ª Edición(2)	-,328	,536	,374	1	,541	,720	,252	2,060
	TNM de 8ªEdición			4,321	2[a]	,115			
	TNM de 8ªEdición(1)	-1,014	,517	3,847	1	,050	,363	,132	,999
	TNM de 8ªEdición(2)	-1,043	,659	2,503	1	,114	,352	,097	1,283
Step 2	pT de 7ª Edición			,405	2	,817			
	pT de 7ª Edición(1)	,190	,308	,380	1	,538	1,209	,661	2,211
	pT de 7ª Edición(2)	,185	,355	,273	1	,602	1,203	,601	2,411
	pT de 8ª Edición			6,731	2	,035			
	pT de 8ª Edición(1)	,876	,339	6,665	1	,010	2,402	1,235	4,673
	pT de 8ª Edición(2)	,581	,478	1,474	1	,225	1,787	,700	4,563
	pN de 7ª Edición	-,947	,554	2,918	1	,088	,388	,131	1,150
	TNM de 7ª Edición			3,988	2[a]	,136			
	TNM de 7ª Edición(1)	-,939	,507	3,429	1	,064	,391	,145	1,056
	TNM de 7ª Edición(2)	-,328	,536	,374	1	,541	,720	,252	2,060
	TNM de 8ªEdición			4,549	3[a]	,208			
	TNM de 8ªEdición(1)	-1,014	,517	3,847	1	,050	,363	,132	,999
	TNM de 8ªEdición(2)	-1,043	,659	2,503	1	,114	,352	,097	1,283
	TNM de 8ªEdición(3)	-,070	,307	,052	1	,819	,932	,511	1,700
Step 3	pT de 8ª Edición			6,438	2	,040			
	pT de 8ª Edición(1)	,850	,337	6,367	1	,012	2,340	1,209	4,529
	pT de 8ª Edición(2)	,558	,478	1,367	1	,242	1,748	,685	4,457
	pN de 7ª Edición	-,813	,511	2,528	1	,112	,444	,163	1,208
	TNM de 7ª Edición			4,489	2[a]	,106			
	TNM de 7ª Edición(1)	-,863	,493	3,058	1	,080	,422	,160	1,110
	TNM de 7ª Edición(2)	-,191	,441	,187	1	,666	,826	,348	1,962
	TNM de 8ªEdición			4,695	3[a]	,196			
	TNM de 8ªEdición(1)	-1,010	,516	3,834	1	,050	,364	,132	1,001
	TNM de 8ªEdición(2)	-1,058	,660	2,567	1	,109	,347	,095	1,266
	TNM de 8ªEdición(3)	-,096	,300	,102	1	,749	,909	,505	1,636
Step 4	pT de 8ª Edición			3,902	2	,142			
	pT de 8ª Edición(1)	,445	,245	3,310	1	,069	1,561	,966	2,521
	pT de 8ª Edición(2)	,070	,318	,049	1	,825	1,073	,575	2,002
	pN de 7ª Edición	-,468	,386	1,471	1	,225	,626	,294	1,334
	TNM de 7ª Edición			8,395	2[a]	,015			
	TNM de 7ª Edición(1)	-1,257	,461	7,428	1	,006	,284	,115	,703
	TNM de 7ª Edición(2)	-,501	,424	1,397	1	,237	,606	,264	1,391
Step 5	pT de 8ª Edición			3,902	2	,142			
	pT de 8ª Edición(1)	,445	,245	3,310	1	,069	1,561	,966	2,521
	pT de 8ª Edición(2)	,070	,318	,049	1	,825	1,073	,575	2,002
	TNM de 7ª Edición			9,229	3	,026			
	TNM de 7ª Edición(1)	-1,257	,461	7,428	1	,006	,284	,115	,703
	TNM de 7ª Edición(2)	-,501	,424	1,397	1	,237	,606	,264	1,391
	TNM de 7ª Edición(3)	-,468	,386	1,471	1	,225	,626	,294	1,334
Step 6	TNM de 7ª Edición			7,537	3	,057			
	TNM de 7ª Edición(1)	-,978	,430	5,168	1	,023	,376	,162	,874
	TNM de 7ª Edición(2)	-,280	,401	,488	1	,485	,755	,344	1,659
	TNM de 7ª Edición(3)	-,215	,359	,360	1	,548	,806	,399	1,629

a. Degree of freedom reduced because of constant or linearly dependent covariates

b. Constant or Linearly Dependent Covariates S = Stratum effect. pN de 7ª Edición = TNM de 7ª Edición(3) + S ;
